# Dilated cardiomyopathy is a part of the *ARV1*-associated phenotype: a case report

**DOI:** 10.1186/s13256-022-03291-0

**Published:** 2022-02-28

**Authors:** Anton Karabinos, Michaela Hyblova, Miroslava Eckertova, Erika Tomkova, Drahomira Schwartzova, Nikoleta Luckanicova, Gabriela Magyarova, Gabriel Minarik

**Affiliations:** 1 Medirex, a.s., Magnezitarska 2/C, 04013 Kosice, Slovakia; 2 Medirex Group Academy n.p.o., Novozamocka 67, Nitra, Slovakia; 3 Medirex, a.s., Galvaniho 17/C, 82016 Bratislava, Slovakia; 4 Pediatric Cardiology Ambulance, Komenskeho 37/A, 04001 Kosice, Slovakia

**Keywords:** ARV1, Dilated cardiomyopathy, Developmental and epileptic encephalopathy, Genetics, Seizures

## Abstract

**Background:**

ACAT-related enzyme 2 required for viability 1 (*ARV1*) encodes a transmembrane lipid transporter of the endoplasmic reticulum, which is presented in all eukaryotes and in plants. Deficiency of *ARV1* is clinically presented as autosomal recessive developmental and epileptic encephalopathy 38 (DEE38) in humans and in mice. So far, three different homozygous and two compound heterozygous *ARV1* mutations in humans have been reported in 15 children.

**Case presentation:**

In this case report we present a novel homozygous *in-frame ARV1*-deletion (c.554_556delTAT, p.L185del) in a 21-year old Caucasian man with developmental delay, intellectual disability, seizures, walking and speech impairments, as well as with a dilated cardiomyopathy (DCM), which has not yet been firmly related to the *ARV1*-associated phenotype. Interestingly, this novel variant lies in the proximity of the p.G189R mutation, which was previously described in two brothers with DEE38 and dilated cardiomyopathy.

**Conclusion:**

The finding of dilated cardiomyopathy in the presented as well as in three previously reported patients from two different families indicates that dilated cardiomyopathy is a part of the *ARV1*-induced DEE38 phenotype. However, more data are needed to make this conclusion definitive.

## Background

**Fig. 1:**
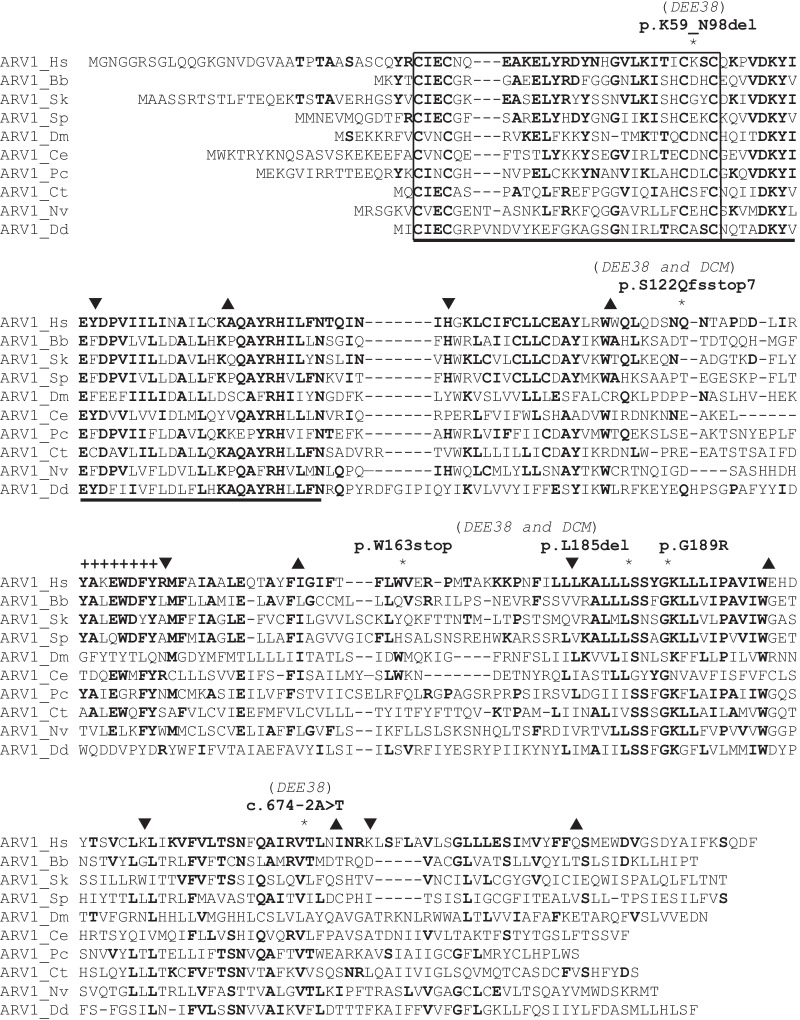
Alignment of the human ARV1 protein with nine homologous ARV1 sequences from different metazoan species and from the slime mold *Dictyostelium* (Amoebozoa). Identical amino acids are marked in bold, while dashes are used to optimize the sequence alignment. The putative zinc-binding motif is boxed, while the proposed N-terminal AHD domain is underlined. Arrowheads pointing down and up mark the beginning and the end, respectively, of the six predicted transmembrane domains (TMD) [1]. The positions of the five previously reported homozygous (p.G189R, p.K59_N98del and c.674-2A>T) and compound heterozygous (p.S122Qfsstop7/p.W163stop) ARV1 variants, as well as the here-presented novel homozygous p.L185del ARV1 variant, are indicated by asterisks. Note that the splice p.K59_N98del variant results in deletion of half of the ADH domain (from K59 to N98), while the second splice c.674-2A˃T variant results in deletion of the entire C-terminal ARV1 sequence V225 downward. As indicated in the picture, all these variants were found in patients with DEE38, while four of them were found in four patients, which, in addition, also revealed DCM (see text for details). Note also that ADH and the first five predicted TMD reveal significant evolutionary conservation, while the rest of the ARV1 sequence and the last predicted TMD reveal only very limited evolutionary conservation (except the nine residues marked by the “+” characters). The sequences shown have the following GenBank accession numbers: ARV1_Hs (*Homo sapiens*, AAG47671.1), ARV1_Bb (*Branchiostoma belcheri*, XP_019640103.1), ARV1_Sk (*Saccoglossus kowalevskii*, XP_006817431.1), ARV1_Sp (*Strongylocentrotus purpuratus*, XP_787555.2), ARV1_Dm (*Drosophila melanogaster*, NP_730651.1), ARV1_Ce (*Caenorhabditis elegans*, NP_001369870.1), ARV1_Pc (*Priapulus caudatus*, XP_014663151.1), ARV1_Ct (*Capitella teleta*, ELU15651.1), ARV1_Nv (*Nematostella vectensis*, XP_032242188.1), ARV1_Dd (*Dictyostelium discoideum*, XP_635839.1).

The ACAT-related enzyme 2 required for viability 1 (*ARV1*) gene encodes a transmembrane lipid transporter of the endoplasmic reticulum, which is conserved in various kingdoms of life. The human *ARV1* encodes a ubiquitously expressed 271-amino-acid protein containing the N-terminal ARV1 homology domain (AHD), which also includes a putative zinc-binding motif and the first of six predicted transmembrane domains (Fig. 1). *ARV1* deletion in yeast results in defects of growth, viability, sterol trafficking, sphingolipid synthesis, membrane organization, telomere organization, hypersensitivity to fatty acids, and glycosylphosphatidylinositol flow (for review, see [1–3] and references therein). Deficiency of ARV1 is clinically presented as autosomal recessive developmental and epileptic encephalopathy 38 (DEE38) in humans and in mice; however, the muscle histology of ARV1 patients, as well as of *ARV1*-knockout mice, indicated that at least skeletal muscle impairment is also a part of the *ARV1*-associated phenotype [[4]; see also the Online Mendelian Inheritance in Man (OMIM) database issue 617020]. So far, three different homozygous *ARV1* and two compound heterozygous mutations in humans have been reported in 15 children (Table 1, Fig. 1). Alazami *et al.* [5] and Palmer *et al.* [4] reported the homozygous missense *ARV1* p.G189R variant in three related patients with developmental delay, severe intellectual disability (ID), infantile-onset epileptic encephalopathy (EE), ataxia, and, in one of them, visual impairment. One of these patients died at the age of 4 years. Palmer *et al.* [4] also reported one additional patient with the homozygous *ARV1* p.K59_N98del splice variant who showed severe neurodevelopmental delay, an intractable infantile onset seizure, movement disorder, and retinal dystrophy and died at the age of 1 year. Davids *et al.* [6] described seven additional children from two different families with global developmental delay, early-onset epilepsy, profound hypotonia, and cortical blindness, while one of them also revealed coarse facial features, hearing loss, and skeletal dysplasia. Two siblings from one family, aged 3 and 17, had a homozygous *ARV1* c.674-2A>T splice variant, which induced the skipping of the last exon 5, encoding a hypervariable C-terminal sequence of ARV1, and a reduction of the expression of truncated protein. The other five members of the second family had the previously described homozygous *ARV1* p.K59_N98del splice variant, which induced the skipping of exon 2, encoding 40 amino acids of the conserved N-terminal ADH domain and a reduction of the expression of the truncated ARV1 protein (see also Fig. 1 for details). All five children carrying the latter variant died by the age of 5 years. Recently, Segel *et al.* [7] presented two brothers aged 11 and 18 with the previously described homozygous *ARV1* p.G189R missense variant and with severe ID, seizures, and autistic regression. Both these patients had no obvious ophthalmologic abnormalities, showed a milder neurocognitive phenotype compared with the patients with the p.K59_N98del splice mutation, described above, and had dilated cardiomyopathy (DCM), which has not been related to the *ARV1*-associated phenotype. Finally, most recently, Darra *et al.* [8] reported two sisters exhibiting severe axial hypotonia, visual inattention, dyskinetic movements, severe developmental delay, and infant epilepsy with migrating focal seizures and myoclonic status. Both these sisters were compound heterozygous for the two p.S122Qfsstop7 and p.W163stop loss-of-function *ARV1* mutations and died by the age of 3 and 9 years. Interestingly, the older girl was also affected by DCM, as were two brothers, documented above, but only three patients from two different families were clearly not sufficient to firmly integrate DCM into the *ARV1*-associated phenotype.

**Table 1:** Some genetic and clinical data of 16 currently known patients with *ARV1*-associated disease

*ARV1* mutation	Mutation type	Patients	Patients with DEE38 (%)	Patients with DCM (%)	Patients who died (%)	References
p.G189R	ho/missense	5	5 (100)	2 (40)	1 (20)	[4, 5, 7]
p.K59_N98del	ho/splice	6	6 (100)	0 (0)	6 (100)	[4, 6]
c.674-2A>T	ho/splice	2	2 (100)	0 (0)	0 (0)	[6]
p.S122Qfsstop7/	ch/loss of	2	2(100)	1 (50)	2 (100)	[8]
p.W163stop	function					
p.L185del	ho/deletion	1	1 (100)	1 (100)	0 (0)	This study
Total (%)	–	16 (100)	16 (100)	4 (25)	9 (56)	–

Note that all patients reveal a neurocognitive DEE38 phenotype, while only 25% of them also reveal, in addition, DCM. Also note that all patients with the p.K59_N98del and p.S122Qfsstop7/p.W163stop mutations died prematurely, in contrast to only one from the rest of the patients (see the text)

*ho* homozygous; *ch* compound heterozygous

In this case report, we present a novel homozygous three-base-pair deletion leading to loss of one amino acid in ARV1 (c.554_556delTAT, p.L185del) in a 21-year-old man with developmental delay, ID, seizures, walking and speech impairments, and DCM.

## Case presentation

The 19-year-old Caucasian patient (proband) came to our genetic consulting service at Medirex/Košice with his formally nonconsanguineous Caucasian parents from a small city in the north of Slovakia. However, a more detailed inspection of the family history revealed that both the parents’ families have their roots in a small village in southern Poland, close to the border with Slovakia. The proband was delivered at term with normal birth parameters (weight 3700 g, body length 52 cm, Apgar scores 9/10/10). His psychomotor milestones were delayed—he started walking at the age of 2 years; presently, at 21 years, he has a vocabulary restricted to about five words, understands simple instructions, is toilet trained, performs simple tasks independently, and attends a special-needs school. His gait is paretic and sometimes ataxic. Epilepsy started at the age of 1 year, and the last seizure of the proband was observed at 17 years. All electroencephalogram profiles performed so far were without lateralization, focal epileptiform activity, or specific graphoelements. Initial brain computerized tomography and magnetic resonance imaging (MRI) revealed cerebellar atrophy and a nonprogressive pituitary microadenoma, while the last MRI at 20 years of age documented a pituitary microadenoma without any configuration/size abnormalities of the cortex, cerebellum, and the brainstem. An echocardiogram at 19 years documented DCM with the left ventricular ejection fraction of 20%; however, no obvious etiology of DCM can be found. An eye examination revealed hypermetropia, hearing was normal, and a standard metabolic investigation was negative. The proband’s parents and his 23-year-old sister were healthy.

**Fig. 2:**
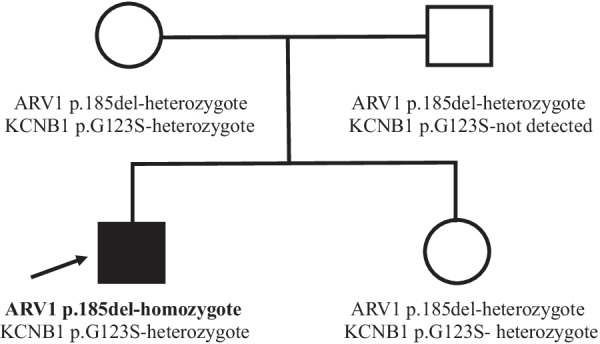
*ARV1* and *KCNB1* mutations in the proband and his family. The proband is indicated by an arrow (see text for details)

Cytogenetic analysis in the proband, performed in another laboratory, revealed a physiological karyotype, while array-based comparative genomic hybridization (aCGH; performed using Agilent SurePrint HD 4x44 platform) did not reveal any gross chromosomal abnormalities. Whole-exome sequencing (Illumina NextSeq550 system; Illumina, San Diego, CA, USA) was performed using the Twist Human Core Exome (Twist Bioscience, San Francisco, CA, USA). Variant calling was conducted on the resulting binary alignment map (BAM) files using Deepvariant [9]. After filtering, the final analysis yielded two rare variants originally classified as variants of uncertain significance, which we considered as potentially causal in context of the proband’s neurocognitive phenotype. The first one was an homozygous in-frame deletion c.554_556delTAT (p.L185del) variant in the *ARV1* gene (allele frequency in the genome aggregation database gnomAD 0.001%); deficiency of ARV1 is associated with the autosomal recessive DEE38 (for details, see “Background” and OMIM 617020). The second one was a heterozygous missense c.367G>A (p.G123S) variant in the *KCNB1* (potassium channel, voltage-gated, Shab-related subfamily, member 1) gene (not listed in gnomAD); *KCNB1-*deficiency is associated with the autosomal dominant DEE26 and includes variable types of seizures late in infancy or in the first years of life, developmental delay with ID, poor speech, and behavioral abnormalities (for details, see OMIM 616056). Sanger sequencing confirmed this result and also revealed that the proband’s parents and sister were heterozygous for the *ARV1* variant, while the proband’s mother and sister (but not father) possessed the same heterozygous *KCNB1* variant (Fig. 2). These results, therefore, suggest that the *KCNB1* mutation, presented also in the healthy mother and sister, most likely represents a benign condition and is not causal for the proband’s phenotype. On the other hand, the *ARV1* variant most likely represents a pathogenic variant, which is causal for the DEE38 disease in the proband. However, using a genetic approach, described above, we were not able to detect any obvious genetic candidate for DCM (for review about DCM, see for example [10]) in the proband.

## Discussion and conclusion

Here we present a 21-year-old patient with developmental delay, ID, seizures, walking and speech impairments, and DCM who possesses a novel homozygous variant (c.554_556delTAT, p.L185del) in the *ARV1* gene. This mutation produces an in-frame deletion of leucine at the highly conserved hydrophobic position 185 of ARV1, which is located in the predicted fourth transmembrane domain, evolutionarily conserved from the slime mold *Dictyostelium* (Amoebozoa) to humans (see Fig. 1 and the corresponding legend for details). Interestingly, this novel variant lies in proximity of the p.G189R mutation, which was previously described by Alazami *et al.* [5] and Palmer *et al.* [4] in three related patients, as well as by Segel *et al.* [7] in two brothers (see Table 1 and “Background” for details). Also interestingly, the latter two brothers revealed DCM, as does our proband, presented here.

Thus, the data in literature, listed above, and the presented case report led us to make the following conclusions. First, a clinical course of *ARV1*-associated disease depends on the type of pathogenic (homozygous or compound heterozygous) mutation: patients with loss-of-function variants (p.S122Qfsstop7, p.W163stop) or splice variants involving the evolutionarily highly conserved N-terminal ADH domain (p.K59_N98del) have a more severe clinical phenotype than those with a probably hypomorphic missense variant (p.G189R), a small deletion of the predicted central transmembrane domain (p.L185del ), or a deletion comprising a hypervariable C-terminal ARV1 sequence (c.674-2A>T; see also Table 1 for a short summary). Second, ARV1-associated disease is primarily a neurodevelopmental condition involving various types of early-onset seizures, developmental delay, ID, hypotonia, premature death, visual impairments, spasticity, and abnormal movements (commonly referred to as DEE38; see above), but the finding of DCM in the presented as well as in three previously reported patients from two different families, described above, indicates that this serious myocardial condition is also a part of the *ARV1*-associated phenotype. However, more data are needed to make this conclusion definitive. Third, a phenotypic heterogeneity, even within members of one family, clearly characterized this newly defined neurodevelopmental and probably also a myocardial *ARV1*-associated disorder. Fourth, the middle part of ARV1 (that is, the positions 185 or 189 of the conserved fourth transmembrane domain, mutated in three out of four currently documented patients with DCM; see text above) might play an important role in the myocardial physiology, but clearly more clinical and molecular analyses are needed to explore this conclusion in more detail.

## Data Availability

The datasets used and/or analyzed during the current study are available from the corresponding author on reasonable request.

## Abbreviations

aCGH: Array-based comparative genomic hybridization
ADH: ARV1 homology domain
ARV1: ACAT-related enzyme 2 required for viability 1
BAM file: Binary alignment map file
DCM: Dilated cardiomyopathy
DEE: Developmental and epileptic encephalopathy
EE: Epileptic encephalopathy
gnomAD: Genome aggregation database
ID: Intellectual disability
KCNB1: Potassium channel, voltage-gated, Shab-related subfamily, member 1
MRI: Magnetic resonance imaging
OMIM: Online Mendelian Inheritance in Man
TMD: Transmembrane domain
